# Chimpanzees are not more aggressive than bonobos but target sexes differently

**DOI:** 10.1126/sciadv.adz2433

**Published:** 2026-03-11

**Authors:** Emile Bryon, Tom S. Roth, Jonas R. R. Torfs, Marcel Eens, Edwin J. C. van Leeuwen, Nicky Staes

**Affiliations:** ^1^ Animal Behaviour and Cognition, Department of Biology, Utrecht University, Padualaan 8, 3584 CA Utrecht, Netherlands.; ^2^ Behavioural Ecology and Ecophysiology Group, Department of Biology, University of Antwerp, Universiteitsplein 1, 2610 Wilrijk, Belgium.; ^3^ Centre for Research and Conservation, Royal Zoological Society of Antwerp, Koningin Astridplein 26, 2018 Antwerp, Belgium.; ^4^ Laboratory for Applied Microbiology and Biotechnology, Department of Bioscience Engineering, University of Antwerp, Groenenborgerlaan 171, 2020 Antwerp, Belgium.; ^5^ Department for Comparative Cultural Psychology, Max Planck Institute for Evolutionary Anthropology, Deutscher Platz 6, 04103 Leipzig, Germany.

## Abstract

The long-standing view that bonobos (*Pan paniscus*) are peaceful while chimpanzees (*Pan troglodytes*) are aggressive has shaped our understanding of primate and human social evolution. However, recent observations from the wild challenge this dichotomy, warranting standardized comparative analyses of aggression in the *Pan* species. Here, we examined aggressive interactions across 22 zoo-housed groups of chimpanzees (*N* = 9 groups, 101 individuals) and bonobos (*N* = 13 groups, 88 individuals) using Bayesian social network analysis. We find no species differences in overall or contact aggression rates, accounting for group size and sex ratio. However, aggression patterns diverge by sex: Bonobos exhibit higher female-to-male aggression, while chimpanzees show the reverse. Notably, absolute aggression rates varied substantially between groups within each species, reinforcing recent evidence on group-specific social structures in *Pan*. These findings challenge the traditional aggression dichotomy between bonobos and chimpanzees and provide insights into the evolutionary dynamics of social conflict strategies in great apes, including humans.

## Teaser

Chimpanzees and bonobos differ in aggression distribution but not magnitude.

## INTRODUCTION

Aggression is a fundamental aspect of human behavior, shaping both individual interactions and broader societal dynamics across cultures (*1*). Unraveling its evolutionary origins is essential for understanding its functions and consequences. Comparative analyses of aggression in nonhuman animals (henceforth “animals”) provide crucial insights into its adaptive significance (*2*–*5*). Among nonhuman primates (henceforth “primates”), aggression serves as a strategic tool for securing vital resources such as territory, food, and mates (*2*, *6*). While aggression is widespread across species, its expression varies considerably depending on socioecological factors (*7*). Comparative studies of closely related species, such as chimpanzees (*Pan troglodytes*) and bonobos (*Pan paniscus*), offer a unique lens through which to examine the evolutionary pressures shaping social behavior in humans. Despite their close phylogenetic relationship, these species exhibit notable differences in aggression (*8*–*11*). As key models for understanding human evolution, assessing the extent of their behavioral divergence is essential for refining our comprehension of aggression in our own species.

Chimpanzees are frequently characterized as relatively violent, engaging in intergroup conflicts with potentially lethal outcomes (*10*, *12*–*17*), whereas bonobos are traditionally perceived as more peaceful, exhibiting higher levels of intergroup tolerance (*10*, *16*, *18*–*22*). In addition, chimpanzees engage in extreme forms of within-group aggression, including coalitionary kills and infanticide, which to date have not been reported for bonobos (*10*, *23*–*28*). In conjunction, these differences have contributed to a portrayal of “peaceful” bonobos and “warlike” chimpanzees (*8*, *10*, *19*, *29*–*31*).

These contrasting patterns have been attributed to ecological differences between bonobos and chimpanzees, both species being separated by the Congo River. Irregular food distribution in chimpanzee habitats promotes higher inter- and intragroup competition, reinforcing male-dominated hierarchies (*32*). In contrast, bonobo habitats feature abundant and evenly distributed food resources, facilitating reduced feeding competition and female cohesion (*33*, *34*). In addition, and unlike chimpanzees, bonobos do not have to compete with gorillas (*Gorilla gorilla*) for access to resources, allowing them to evolve in stable, less competitive environments that could support their unique social dynamics (*31*). Last, the lower predation pressure in bonobo habitats may have reduced the need for male-dominated group defense, enabling females to attain higher social status (*35*). Consequently, the Congo River’s role as a geographic barrier further facilitated divergent evolutionary trajectories in each species (*31*, *36*).

In bonobos, these environmental circumstances likely encouraged female cooperation and support, enabling selection against male aggression—a process referred to as “self-domestication.” According to this theory, female preference for less aggressive males led to reduced overall aggression in bonobos compared to chimpanzees (*31*, *36*). This mechanism has been suggested to have shaped our species’ evolution, where selection against aggression may have facilitated increased prosociality and complex social structures (*37*–*39*). A parallel “selection against aggression” process in one of two species that are most closely related to ours is a decisive conjecture when tracing the evolutionary roots of aggression in humans, as it suggests that similar evolutionary pressures and evolutionary dynamics could have characterized the early steps of our own speciation. However, aggression remains a key behavioral strategy for securing resources, and species-specific differences in dominance structures and sex-based aggression patterns remain to be examined in a detailed fashion. In bonobos, female coalitions suppress male aggression and enable female dominance (*10*, *34*), whereas in chimpanzees, males use coercion to assert dominance over females (*40*, *41*). Aggression also seems to vary as a function of sexes: Bonobo females compete with each other for mates and social status more than chimpanzee females (*42*, *43*), while bonobo males exhibit aggressive behavior toward other males similar in frequency (*42*) but lower in severity (i.e., resulting in physical contact) than their chimpanzee counterparts (*44*).

Despite widespread assumptions about bonobo pacifism and chimpanzee aggression, systematic comparative studies using standardized methodologies in both species remain scarce. Recent comparative research conducted in the wild reports conflicting results. In line with the dominant view on *Pan* species aggression tendencies, research focused on male aggression patterns shows higher contact aggression in Kalinzu (Uganda) chimpanzee males (group M, *N* = 10) than Wamba (Democratic Republic of the Congo) bonobo males (group E1, *N* = 11) (*44*). However, another study challenges previous generalizations, reporting that wild Kokolopori (Democratic Republic of the Congo) bonobo males (three groups, *N* = 12) exhibit approximately three times the aggression rates of Gombe (Tanzania) chimpanzee males (two groups, *N* = 15), even when limiting analyses to physical contact aggression (*45*). The latter is especially unexpected given the higher rates of lethal aggression and thus likely also sublethal aggression rates in chimpanzees compared to bonobos (*10*, *41*, *44*). These results warrant a reevaluation of the role of aggression in bonobo social dynamics (*11*, *46*) and highlight the importance of studies that control for ecological and methodological differences. In addition, such studies hold the potential to corroborate different results from wild populations by drawing from a larger sample size, aiming to detect (potential) species-wide effects. Moreover, while Mouginot *et al.* (*45*) compare male aggression using dyadic rates, these insights call for similar analyses of female strategies and individual aggression rates to capture the prevalence and sex-based distribution of aggression.

To further investigate species and sex-specific aggression patterns, we present a large-scale systematic comparison of 22 groups of zoo-housed *Pan* apes (*N* = 189, 110 chimpanzees and 88 bonobos). This multigroup approach accounts for behavioral plasticity reported in both *Pan* species (*47*–*49*) and removes many of the confounding ecological variables present in wild populations (*7*, *10*). As such, potential differences in *Pan* aggression could be more confidently attributed to hardwired adaptive species-level effects shaped by evolutionary pressures with a potential genetic and/or neurological basis, rather than a consequence of direct environmental pressures. By examining both dyadic and individual aggression with respect to total and contact aggression, we aim to refine our understanding of inter- and intrasex aggression across species and explore potential evolutionary mechanisms underlying these behaviors. We investigate dyadic aggression using Bayesian social network analyses, which account for the social context and relationships that shape aggressive interactions. By capturing complex dependencies within social networks, these models yield more insightful inferences. In addition, they offer full posterior distributions rather than point estimates and enable posterior predictive checks to evaluate the robustness of results. We also conduct additional analyses by comparing results across samples using different age cutoff points (7 and 12 years old), reflecting the range commonly found in the literature (*44*, *45*, *50*, *51*). Doing so, we engage in statistical power/life stage inclusion trade-offs, allowing us to remain flexible in scope while aiming to capture reliable and representative patterns of aggression in *Pan* species. Last, we also examine group differences in aggression to shed further light on behavioral plasticity between and within species, in line with recent research showing underestimated overlap in social behaviors in the *Pan* species (*52*) and variation in aggression patterns from wild populations (*44*, *45*). Our findings contribute to the broader discussion of the role of aggression in primate evolution and provide a comparative framework for understanding the evolutionary underpinnings of human aggression (*35*, *53*).

## RESULTS

Across the 22 groups, 3243 instances of directed aggression by and toward individuals above 7 years old were observed (1368 from bonobos and 1875 from chimpanzees), of which 1193 were contact aggressions (456 from bonobos and 737 from chimpanzees). Including only aggressors and targets aged above 12 years results in 2127 instances of directed aggression (558 from bonobos and 1569 from chimpanzees), of which 833 were contact aggression (171 from bonobos and 662 from chimpanzees).

### Individual-level total aggression

**Fig. 1.**
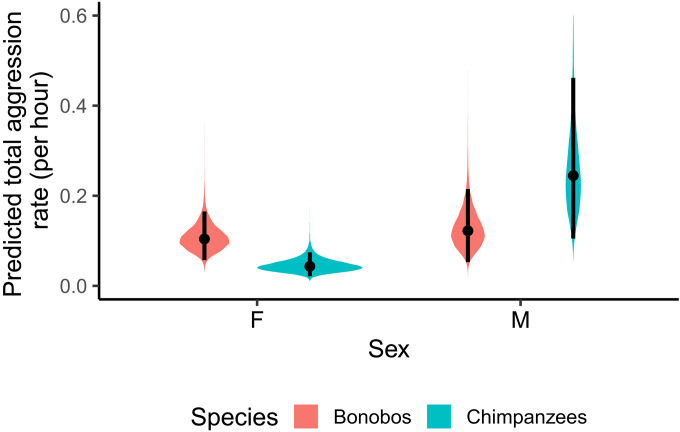
The density plots depict the posterior distributions for the effect of both sex and species on the predicted total aggression rate. Black lines reflect a 95% CI.

First, we modeled individual counts of aggression as a function of species and sex while controlling for group ID and observation time through an offset term (table S1). Averaged across males and females, our results provided weak-to-moderate evidence that our data aligned better with a null model, suggesting no overall difference in aggression rate between species [*b* = 0.04; 95% credible interval (CI): [−0.36, 0.43]; probability of direction (pd) = 0.597; BF01 = 2.68–5.10–7.43]. In addition, averaged over both species, males were more aggressive than females (*b* = −0.47; 95% CI: [−0.71, −0.22]; pd = 0.999; BF10 = 44.53–32.85–26.37). However, this effect was mainly driven by chimpanzee males, as aggression rates within species were modulated by sex. We found strong evidence for a robust interaction between sex and species (*b* = 0.39; 95% CI: [0.16, 0.63]; pd = 0.999; BF10 = 23.59–13.70–18.82; Fig. 1).

Exploring this interaction, we found very strong evidence for a sex difference in aggression in chimpanzees, with males being more aggressive than females (ΔF-M = −1.74; 95% CI: [−2.43; −1.04]; pd = 1.000; BF10 = all >100). For bonobos, we found moderate-to-strong evidence for the null model, suggesting that male and female bonobos show equal amounts of total aggression counts (ΔF-M = −0.16; 95% CI: [−0.81; 0.52]; pd = 0.687; BF10 = 3.83–7.89–11.21). When comparing between species, we found mixed evidence for a species difference in female aggression, although bonobo females were slightly more aggressive than chimpanzee females (Δbonobo-chimp = 0.88; 95% CI: [0.10; 1.74]; pd = 0.979; BF10 = 3.18–1.41–0.92), and weak evidence against a species difference in male aggression (Δbonobo-chimp = −0.69; 95% CI: [−1.74; 0.28]; pd = 0.922; BF01 = 1.12–2.08–3.04).

Thus, our results indicate that bonobos do not systematically differ from chimpanzees in total aggression rates. In chimpanzees, males are on average more aggressive than females, while male and female bonobos show similar aggression rates that, on average, fall intermediate to rates of male and female chimpanzees.

When using an age cutoff of 12 years old, the patterns were largely similar (table S2). We found weak evidence against a species difference in aggression (*b* = −0.34; 95% CI: [−0.90, 0.22]; pd = 0.892; BF01 = 1.13–1.61–2.40) and strong evidence for males being more aggressive than females overall (*b* = −0.56; 95% CI: [−0.87, −0.25]; pd = 0.999; BF10 = 53.15–67.74–26.74). This difference tended to be more extreme in chimpanzees, although the Bayes factors (BFs) are inconclusive (*b* = 0.29; 95% CI: [−0.01, 0.59]; pd = 0.970; BF01 = 1.93–1.04–0.76).

### Individual-level contact aggression

**Fig. 2.**
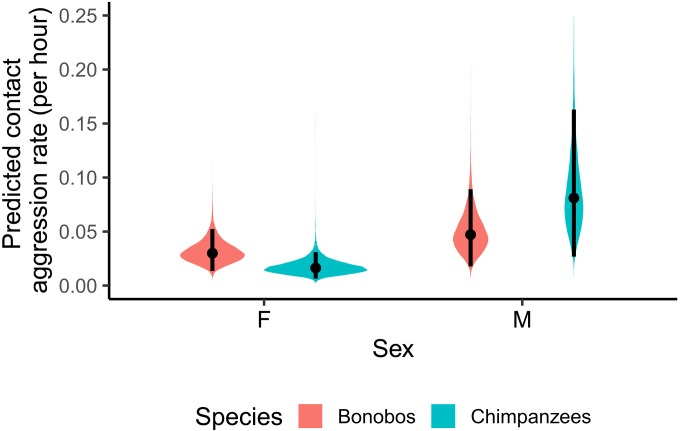
The density plots depict the posterior distributions for the effect of both sex and species on the predicted contact aggression rate. Black lines reflect a 95% CI.

Next, we investigated whether similar results hold when only focusing on physical “contact” aggression. We modeled individual counts of contact aggression as a function of species and sex while controlling for group ID and observation time through an offset term (table S3). Again, when averaged across males and females, our results provided weak-to-moderate evidence that our data aligned substantially better with a null model, suggesting no species-wide differences in contact aggression rate (*b* = 0.02; 95% CI: [−0.43, 0.46]; pd = 0.527; BF01 = 2.47–4.77–6.67). Furthermore, we found moderate-to-strong evidence for a sex effect when averaged over both species, with males showing more contact aggression than females (*b* = −0.52; 95% CI: [−0.80; −0.21]; pd = 0.999; BF10 = 24.44–16.97–11.51). Different from the total aggression analysis, the BF here indicated inconclusive evidence for an interaction between sex and species, and the CI slightly overlapped zero (*b* = 0.29; 95% CI: [−0.01; 0.58]; pd = 0.973; BF10 = 1.78–1.09–0.84; Fig. 2). This suggests that the interaction is not as robust as in the total aggression analysis, although the pattern is similar, with a sex difference in chimpanzees but not in bonobos.

Limiting our analysis to contact aggression thus revealed similar results to the total aggression analyses, with bonobos and chimpanzees not systematically differing in rates of contact aggression, although males appear to show more contact aggression than females in both species. While some evidence was present for an interaction between sex and species, the scores indicate that the effect is weak, meaning that contact aggression rates of males and females do not clearly differ between bonobos and chimpanzees. The weak interaction seems to be driven by chimpanzee males, who show more contact aggression than chimpanzee females (ΔF-M = −1.62; 95% CI: [−2.43; −0.72]; pd = 0.999; BF10 = 51.99–29.82–17.57), while in bonobos, rates were only slightly higher for males than females (ΔF-M = −0.46; 95% CI: [−1.28; 0.32]; pd = 0.876; BF01 = 1.91–3.43–5.01).

When using an age cutoff of 12 years old, the patterns were somewhat similar (table S4). We found inconclusive evidence regarding a species difference in contact aggression (*b* = −0.47; 95% CI: [−1.11, 0.13]; pd = 0.938; BF01 = 0.72–1.01–1.47) but strong evidence for males showing more contact aggression than females overall (*b* = −0.58; 95% CI: [−0.96, −0.19]; pd = 0.997; BF10 = 15.06–11.37–7.44). We also found inconclusive results regarding the interaction between sex and species (*b* = 0.25; 95% CI: [−0.14, 0.62]; pd = 0.902; BF01 = 1.21–2.01–2.98).

### Dyadic-level total aggression (raw edge weights)

Next, to investigate how individuals of both sexes and species distribute their aggression, we investigated dyadic aggression. We modeled edge weights (i.e., connections between individuals expressed in frequencies of aggression) from the dyadic total aggression networks as a function of species, aggressor sex, and receiver sex while controlling for group ID and individual ID (table S5).

**Fig. 3.**
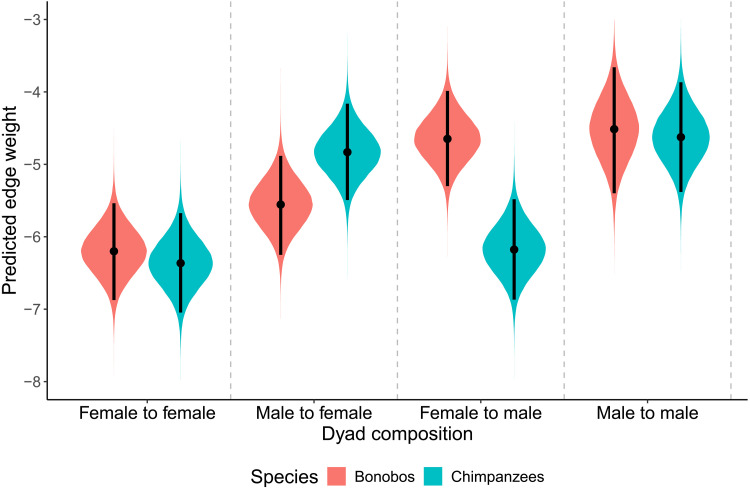
The density plots depict the posterior distributions for the effect of both dyad composition and species on edge weight in the total aggression networks. Black lines reflect a 95% CI.

Averaged across the recipient and aggressor sex combinations (Fig. 3), we found weak-to-moderate evidence against a species difference in aggression (*b* = 0.13; 95% CI: [−0.27, 0.54]; pd = 0.748; BF01 = 1.24–2.00–3.65). In addition, we found weak-to-moderate evidence against a species difference in female-to-female aggression (Δbonobo-chimp = 0.16; 95% CI: [−0.77; 1.10]; pd = 0.636; BF01 = 2.36–4.00–7.65) and male-to-male aggression (Δbonobo-chimp = 0.11; 95% CI: [−1.02; 1.21]; pd = 0.576; BF01 = 2.10–3.47–6.49) and inconclusive evidence regarding a species difference in male-to-female aggression (Δbonobo-chimp = −0.72; 95% CI: [−1.66; 0.19]; pd = 0.939; BF01 = 0.49–1.30–2.95). However, we found strong evidence for a species difference in female-to-male aggression, with edge weights being higher for bonobos than for chimpanzees (Δbonobo-chimp = 1.53; 95% CI: [0.61; 2.46]; pd = 0.999; BF10 = >100–37.92–10.63). In addition, we found robust differences between dyad compositions within each species (table S6). Most notably, we found stronger evidence for differences between dyad compositions in chimpanzees than in bonobos. In chimpanzees, all of these differences were driven by the fact that males were more aggressive than females, irrespective of the sex of the receiver. In bonobos, however, robust differences in edge weights seemed to be explained by receiver sex, with males receiving more aggression than females.

When using an age cutoff of 12 years old (table S7), we found weak-to-moderate evidence against a species difference in dyadic aggression averaged over all sex combinations (*b* = 0.03; 95% CI: [−0.42, 0.48]; pd = 0.555; BF01 = 1.40–2.19–4.09). Furthermore, we found weak-to-moderate evidence against species differences in male-to-male and female-to-female aggression, inconclusive evidence regarding male-to-female aggression, and weak-to-strong evidence for a species difference in female-to-male aggression (table S8). Within species, we still found robust differences between dyad compositions, especially in chimpanzees (table S9).

### Dyadic-level contact aggression (raw edge weights)

**Fig. 4.**
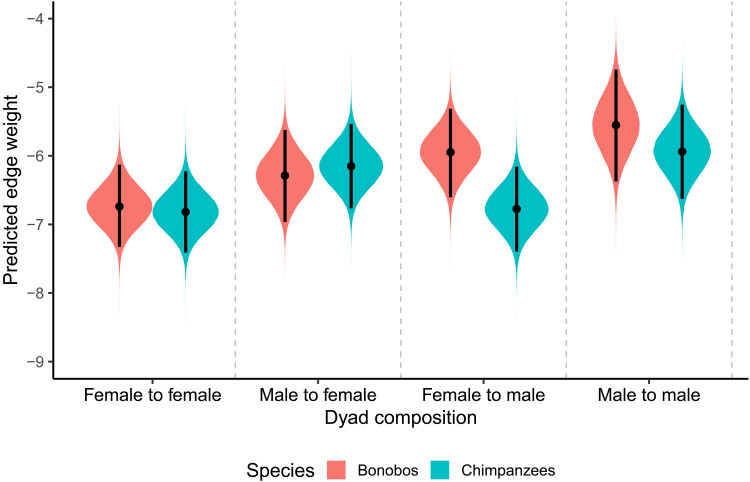
The density plots depict the posterior distributions for the effect of both dyad composition and species on edge weight in the contact aggression networks. Black lines reflect a 95% CI.

To see whether these results hold when limiting our analysis to contact aggression, we modeled edge weights from dyadic contact aggression networks as a function of species, aggressor sex, and receiver sex while controlling for group ID and individual ID (table S10). Averaged across the recipient and aggressor sex combinations (Fig. 4), we found weak-to-moderate evidence against a species difference in aggression (*b* = 0.14; 95% CI: [−0.22, 0.50]; pd = 0.790; BF01 = 1.31–2.03–3.91). We found weak-to-moderate evidence against species differences in male-to-female (Δbonobo-chimp = −0.14; 95% CI: [−1.04; 0.76]; pd = 0.615; BF01 = 2.18–4.17–7.75), female-to-female (Δbonobo-chimp = 0.08; 95% CI: [−0.75; 0.92]; pd = 0.573; BF01 = 2.95–4.61–8.85), and male-to-male (Δbonobo-chimp = 0.39; 95% CI: [−0.62; 1.44]; pd = 0.770; BF01 = 1.76–2.93–5.94) rates of contact aggression. However, we found inconclusive evidence regarding female-to-male aggression, although we observed more female-to-male aggression in bonobos than chimpanzees (Δbonobo-chimp = 0.82; 95% CI: [−0.03; 1.68]; pd = 0.970; BF01 = 0.46–0.76–1.83). Similar to the total aggression analysis, we found robust differences between specific dyad compositions within each species (table S11). The contact aggression contrasts for bonobos are rather inconclusive or weak, while the results for chimpanzees are relatively similar to the total aggression analysis, even though the differences are less outspoken.

When using an age cutoff of 12 years old (table S12), we found weak-to-moderate evidence against a species difference in dyadic aggression averaged over all sex combinations (*b* = 0.01; 95% CI: [−0.40, 0.42]; pd = 0.527; BF01 = 1.56–2.43–4.46). For all dyad compositions, we found weak-to-moderate evidence for the absence of a species difference in contact aggression (table S13). Considering differences between dyad compositions within species, we found some robust differences in chimpanzees but found weak-to-moderate evidence for the absence of differences or inconclusive evidence in bonobos (table S14).

### Dyadic-level total aggression (*z*-scored edge weights)

Last, to examine the relative distribution of aggression, we standardized aggression rates within groups using *z*-scored edge weights, eliminating between-group variation. This ensures no differences in mean aggression rates between groups and species, allowing us to focus on within-group patterns. Unlike the previous analyses using raw edge weights, *z*-scoring accounts for differences in overall aggression intensity and variability, highlighting whether certain aggression types—such as male-to-female aggression—are disproportionately high or low within groups. Thus, by comparing *z*-scored values across groups and species, we do not assess species differences in absolute aggression rates (see previous analyses) but in how aggression is distributed within the apes’ social networks.

**Fig. 5.**
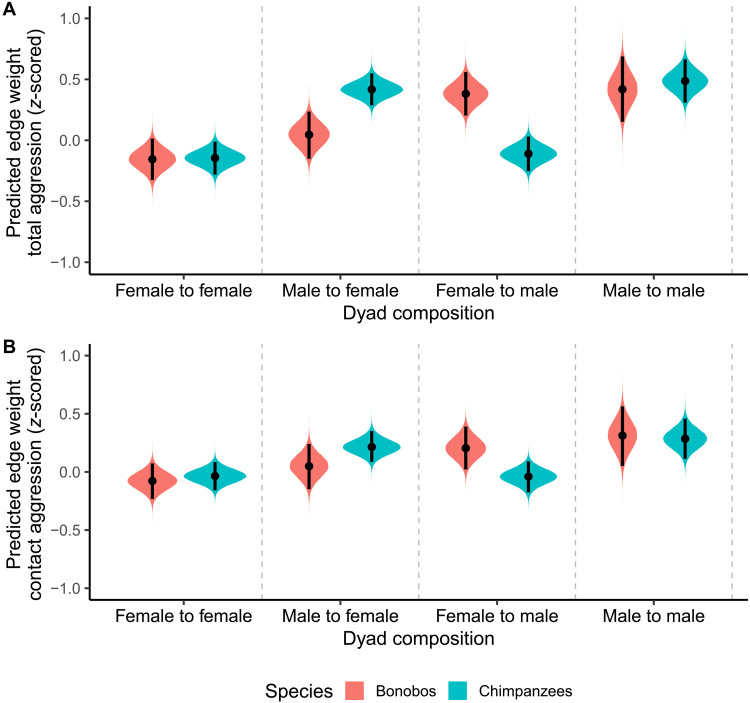
The density plots depict the posterior distributions for the effect of both dyad composition and species on *z*-scored edge weight. (**A**) shows the plots in the total aggression networks, and (**B**) shows the plots in the contact aggression networks. Black lines reflect a 95% CI.

We modeled *z*-scored edge weights from the dyadic total aggression networks as a function of species, aggressor sex, and receiver sex while controlling for group ID and individual ID (table S15). The results with *z*-scored edge weights as a dependent variable strongly mirror the raw edge weight analysis (Fig. 5), although the relative effects of dyad composition became more apparent after removing between-group variation. We found evidence for species differences in the relative occurrence of male-to-female (very strong; Δbonobo-chimp = −0.37; 95% CI: [−0.60; −0.14]; pd = 0.999; BF10 = 79.40–21.07–14.57) and female-to-male total aggression (very strong; Δbonobo-chimp = 0.49; 95% CI: [0.27; 0.71]; pd = 1.000; BF10 = >100–>100–>100), with more male-to-female aggression in chimpanzees than bonobos and more female-to-male aggression in bonobos than chimpanzees. However, we found evidence against a species difference in the relative occurrence of female-to-female (Δbonobo-chimp = −0.01; 95% CI: [−0.22; 0.21]; pd = 0.536; BF01 = 3.89–9.17–18.74) and male-to-male total aggression (Δbonobo-chimp = −0.07; 95% CI: [−0.38; 0.25]; pd = 0.665; BF01 = 2.29–5.77–11.47). Furthermore, we found robust differences in relative occurrence of aggression between dyad compositions within species (table S16), largely mirroring the results for the raw edge weight analysis.

When using an age cutoff of 12 years old (table S17), we found weak-to-moderate evidence for the absence of a species difference in relative occurrence of male-to-male aggression and moderate-to-strong evidence for the absence of a species difference in female-to-female aggression (table S18). Moreover, the evidence regarding male-to-female aggression was inconclusive, while we found strong evidence for a robust species difference in the relative occurrence of female-to-male aggression. Considering differences between dyad compositions within species, we found some robust differences in bonobos but especially in chimpanzees (table S19).

### Dyadic-level contact aggression (*z*-scored edge weights)

Similarly, here, we modeled *z*-scored edge weights from the dyadic contact aggression networks as a function of species, aggressor sex, and receiver sex while controlling for group ID and individual ID (table S20). The results with *z*-scored edge weights as a dependent variable again largely mirror the raw edge weight analysis (Fig. 5). We found moderate-to-strong evidence against a species difference in the relative occurrence of female-to-female contact aggression (Δbonobo-chimp = −0.04; 95% CI: [−0.23; 0.15]; pd = 0.674; BF01 = 4.08–9.21–18.86), weak-to-strong evidence against a species effect in male-to-male contact aggression (Δbonobo-chimp = 0.03; 95% CI: [−0.28; 0.33]; pd = 0.569; BF01 = 2.67–6.32–12.27), and weak-to-moderate evidence against a species difference in male-to-female contact aggression (Δbonobo-chimp = −0.17; 95% CI: [−0.40; 0.07]; pd = 0.921; BF01 = 1.19–3.01–6.03). For female-to-male aggression, we found conflicting results. While the CIs indicate that female-to-male aggression is slightly more common in bonobos, the BFs are inconclusive (Δbonobo-chimp = 0.25; 95% CI: [0.02; 0.47]; pd = 0.986; BF01 = 0.51–0.81–2.39). Again, we found robust differences in the relative occurrence of contact aggression between dyad compositions within species (table S21). Differences within chimpanzees were again more outspoken than within bonobos.

When using an age cutoff of 12 years old (table S22), we found weak-to-moderate evidence for the absence of a species difference in the relative occurrence of contact aggression for male-to-female, female-to-male, and male-to-male dyads and moderate-to-strong evidence for the absence of a difference in female-to-female aggression (table S23). Considering differences between dyad compositions within species, we found some robust differences in chimpanzees but found weak-to-moderate evidence for the absence of differences or inconclusive evidence in bonobos (table S24).

### Group-level differences

**Fig. 6.**
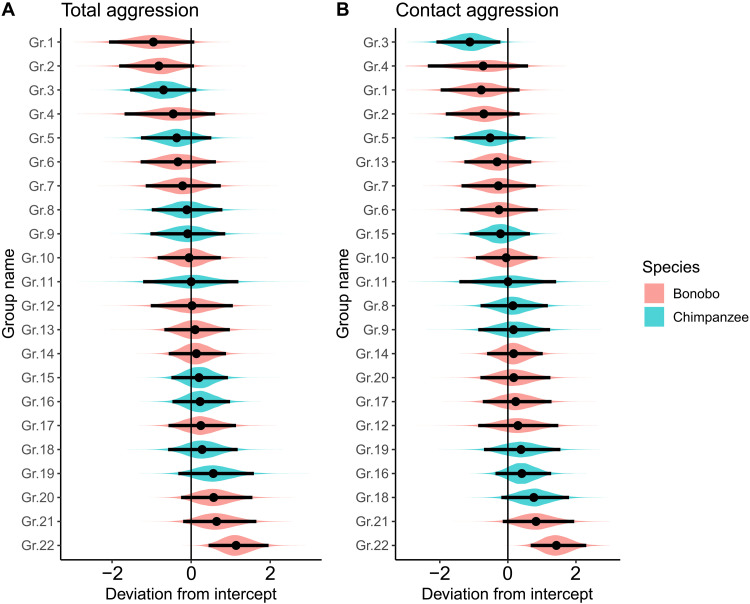
Posterior sample distributions for the group intercepts for aggression. (**A**) shows distribution for total aggression, and (**B**) shows distribution for contact aggression only. Black lines reflect a 95% CI.

We plotted posterior sample distributions for the group intercepts with both total and contact aggression of the individual-level analyses to further illustrate the distribution of aggression within species (Fig. 6). The plots show substantial overlap between groups with both total and contact counts of aggression, with some of the most and least aggressive groups in bonobos (total aggression range: −0.96 to 1.14; contact aggression range: −0.78 to 1.44) and chimpanzees (total aggression range: −0.68 to 0.57; contact aggression range: −1.11 to 0.77).

## DISCUSSION

By systematically examining aggression across 9 chimpanzee groups and 13 bonobo groups using standardized observation protocols, our findings challenge prevailing scientific accounts and popular beliefs. Contrary to the expectation that bonobos exhibit lower levels of aggression than chimpanzees, we found no overall differences in absolute aggression rates between the two species. Excluding individuals under 12 years old yielded patterns mostly consistent with our main findings, although with lower certainty because of the reduced sample size. We interpret this consistency as further evidence for the robustness of our results across age-inclusive and adult-only samples. This result held true for both total aggressive interactions and contact aggression alone, which also differs from research conducted in wild populations. Instead, our results highlight the importance of sex-specific aggression patterns highlighted in wild groups by Mouginot *et al.* (*45*), with bonobos exhibiting more female-to-male aggression and chimpanzees showing more male-to-female aggression, particularly when controlling for group-level variation. Rather than revealing species-wide differences, our results suggest that group identity is a major determinant of aggression rates in *Pan* species, consistent with a recent study on social tolerance (*48*) and suggested by contrasting results coming from different wild groups (*44*, *45*). Our study contributes to ongoing efforts to systematically compare aggression rates and patterns in our closest living relatives. Similar to recent investigations of wild populations (*45*), our findings diverge from traditional species-based generalizations, emphasizing the need for more nuanced interpretations of aggression in *Pan*. Here, we contextualize these results in relation to existing hypotheses concerning the expression and evolutionary origins of aggression in humans and other primates.

The absence of species-level differences in aggression is a notable finding, persisting even when restricting analyses to contact aggression, which arguably represents a more severe form of aggression. While extreme forms of aggression, such as intergroup conflicts and coalitionary killings, are well documented in chimpanzees, such events are rare and do not reliably predict differences in routine within-group aggression reported here. Nonetheless, our findings challenge the notion that aggression is inherently more pronounced in chimpanzees than in bonobos. Despite the absence of species-wide differences, sex remained a key factor in the distribution of aggression. While males exhibited higher aggression levels than females on average, this pattern was driven primarily by chimpanzees, as aggression rates between male and female bonobos largely overlapped. Contact aggression followed a similar pattern, with males in both species displaying more contact aggression than females. Although these findings do not fully align with predictions of the self-domestication hypothesis (i.e., reduced aggression in bonobo males), the sex-based differences in aggression distribution warrant further investigation, particularly in terms of dyadic aggression patterns.

Our findings revealed consistent species-specific patterns in between-sex aggression. Male-to-female aggression was more prevalent in chimpanzees than in bonobos, whereas female-to-male aggression was more pronounced in bonobos, reinforcing the dominant social role of females in bonobo societies. Our results match the recent findings from the wild groups of Kokolopori bonobos (*N* = 3 groups) and Gombe chimpanzees (*N* = 2 groups) (*45*). These patterns align with established social structures, where the dominant, coalitionary sex is more likely to direct aggression toward the subordinate sex (*6*, *42*, *54*, *55*). However, within-sex aggression did not differ substantially between species, regardless of whether total or only contact aggression was considered. The finding that male-to-male (contact) aggression levels were similar in both species differs from recent studies conducted in the wild (*44*, *45*) and contradicts the notion that bonobos have been selected for reduced aggression. While bonobo females have been observed to engage in competition for mates and social dominance, our results did not indicate higher levels of female-to-female aggression compared to female chimpanzees. It remains possible that bonobo female aggression is socially mediated through alternative mechanisms such as sociosexual behaviors or redirected aggression toward males [e.g., see Brooker *et al.* (*52*)]. Further research is needed to explore these possibilities in greater detail.

The self-domestication hypothesis posits that selection against male aggression in bonobos has led to an overall reduction in aggression relative to chimpanzees, with neotenic traits and behavioral markers supporting this process (*31*). However, our findings, in line with those of Mouginot *et al.* (*45*), do not support this hypothesis. Bonobo males exhibited aggression levels comparable to those of chimpanzees, both in total and contact aggression. While the higher incidence of female aggression toward males in bonobos aligns with self-domestication predictions, the persistence of male aggression in both species challenges the idea that selection against aggression is a defining characteristic of bonobo evolution. Our results suggest that aggression remains a central aspect of bonobo social behavior, rather than being systematically reduced as predicted by the self-domestication hypothesis. Instead of an overall reduction in aggression, our findings highlight distinct strategic patterns of aggression: Whereas chimpanzees exhibit male-dominated aggression directed at both sexes, bonobo aggression is more evenly distributed between males and females but disproportionally targets males.

Our findings contribute to a growing body of evidence suggesting that behavioral patterns in *Pan*, including aggression, may be more influenced by group identity than by species-wide traits (*48*, *56*–*58*). This is supported by discrepancies in recent studies of wild populations, which report higher rates of male-to-male aggression in bonobos at Kokolopori compared to chimpanzees at Gombe (*45*) but greater male-to-male contact aggression in chimpanzees at Kalinzu compared to bonobos at Wamba (*44*). Such inconsistencies underscore the importance of large-scale datasets that capture the full variability of aggression within *Pan* species. Our study reinforces this notion by demonstrating substantial group-level variation. For example, the most aggressive groups included two bonobo groups and one chimpanzee group, while the three least aggressive groups were two bonobo groups and one chimpanzee group. Thus, it seems that only by sampling many groups of the same (*Pan*) species, accurate species-level tendencies may be captured (*49*, *59*, *60*).

While acknowledging that findings may differ between wild and captive populations, our study benefits from controlled socioecological environments that allow for direct species comparisons—a feature that may be less equal or comparable among wild field sites. Although captivity may limit certain forms of aggression (e.g., no intergroup conflicts) and generate unusual groups when compared to the wild (in terms of size or in age and sex composition), our data reveal substantial variation in aggression between populations, corroborating results from varied wild populations (*44*, *45*). This suggests that within-group aggression in captivity may still reflect meaningful species-typical behavioral tendencies, as does selective interindividual interaction in great apes in confined spaces more generally (*61*–*63*). For chimpanzees, a systematic analysis of subspecies differences may also be warranted, as prior research suggests that Eastern chimpanzees (*Pan troglodytes schweinfurthii*) exhibit higher aggression levels than Western chimpanzees (*Pan troglodytes verus*) (*27*, *64*). Moreover, coalitionary dynamics also differ between both *Pan* species, as polyadic aggressions often involve all-male aggressors in chimpanzees and all-female or mixed-sex aggressors in bonobos (*41*, *44*, *45*, *65*, *66*). Coalitionary dynamics are therefore also representative of power imbalance between sexes and should be incorporated in future aggression analysis frameworks. In addition, bonobo coalitions appear to disproportionately target males (*66*), further reflecting our own results—most aggression being expressed by males in chimpanzees and targeted at males in bonobos. Therefore, it is warranted to include these patterns to further understand the distribution and dynamics of aggression and dominance in bonobos and chimpanzees. Last, further investigation and novel approaches in captive settings that bear in mind the distinction between proactive and reactive aggression (*39*, *67*) could test hypotheses regarding the functions and triggers of aggression. Although their applicability is debated in humans (*68*, *69*), these types of aggression have been mapped onto the way aggression is expressed and strategized in chimpanzees and bonobos. Such research offers opportunities to further explore the evolutionary functions and roots of aggression in the *Pan* species and in our own.

Our results indicate that within-group aggression rates do not differ substantially between bonobos and chimpanzees, aligning with recent reports from wild populations and challenging long-standing assumptions about bonobo peacefulness. These results call into question the validity of the self-domestication hypothesis and provide fresh insights into the evolutionary origins of human aggression. Rather than being characterized by an overall reduction in aggression, bonobo social systems appear to redistribute aggression in a sex-specific manner. Future research should continue to explore the ecological, genetic, and social factors that shape aggression in *Pan* species, ultimately refining our understanding of the role of aggression in primate and, specifically, human evolution.

## MATERIALS AND METHODS

### Subjects and housing

Behavioral data were collected among 22 groups—13 groups of bonobos and 9 groups of chimpanzees—in 16 different European locations (Table 1) between 2011 and 2024. Housing conditions all included large spaces shared between large indoor and outdoor enclosures. One hundred and eighty-nine adult subjects were included in the analyses, which included 101 chimpanzees and 88 bonobos. We only included individuals who were at least 7 years old or older at the end of the observation period (thereby excluding 50 infants and juveniles), as individuals having reached this age are neurologically, sexually, and socially developed to the point of independent integration into their groups’ social network (*50*, *51*, *62*, *70*–*72*). In addition, sensitivity analyses were conducted by only including individuals aged 12 years and above [similarly to Mouginot *et al.* (*45*)], at which point chimpanzees and bonobos are considered to reach full maturity (*50*, *51*, *73*). Individuals ranged in age from 7 to 71 years old, including 119 females (56 bonobos and 63 chimpanzees) and 70 males (32 bonobos and 38 chimpanzees) (Table 1). The subset of individuals aged 12 years and above comprises 105 females (45 bonobos and 60 chimpanzees) and 57 males (22 bonobos and 35 chimpanzees). The sexes of the apes were derived from the zoos’ studbooks. Last, because of the data collection period spanning 13 years and zoological institutions moving individuals between groups for breeding purposes, two individuals are repeated over two different groups (Banya and Eja, two bonobo females, respectively aged 22.77 in Twycross and 33 in Planckendael and aged 22.56 in Wuppertal and 31.74 in Ouwehands) but are observed with ±10 years in between. Because only these two animals have multiple records, it is not possible to include an individual-level random intercept without poor identifiability. Therefore, for the individual-level analyses, we treated each record as a separate observation. For the dyadic models, we were able to account for repeated measures. Here, the two records per animal were treated as the same underlying individual, as we included a multimembership term for individual ID.

**Table 1.** List of zoos with the number of groups and demographics.

Species	Zoo (year)	Groups	Older than 12	Older than 7	Younger than 7	Sex ratio (>7 only, % female)
Bonobos	Apenheul (2022)	1	8	9	3	66.67
	Frankfurt (2022)	2	5	6	1	83.33
			5	6	4	50
	Ouwehands (2022)	1	7	10	4	60
	Planckendael (2023)	2	6	8	2	37.5
			4	7	4	85.71
	Stuttgart (2022)	3	4	5	3	60
			3	5	4	80
			3	3	3	66.67
	Twycross (2011)	2	4	5	1	80
			3	4	2	50
	Vallée des Singes (2021)	1	11	15	2	66.67
	Wuppertal (2013)	1	4	5	2	40
Chimpanzees	Antwerp (2023)	1	11	11	0	54.55
	Beekse Bergen (2024)	2	10	11	4	63.64
			8	9	3	44.44
	Beauval (2024)	1	11	13	1	61.54
	Bussolengo (2024)	1	7	7	0	85.71
	Dierenrijk (2023)	1	6	7	1	71.43
	Leintal (2024)	1	29	30	2	56.67
	Valencia (2024)	1	6	6	2	83.33
	Wingham (2024)	1	7	7	2	71.43

### Data collection

Data were collected by 16 observers using an ethogram common across both species, developed for bonobos by Stevens and colleagues (*74*). Data on aggression were collected through all occurrence behavior sampling (*75*) of all agonistic interactions among subadult and adult (i.e., >7 years old) individuals of both sexes during daily observations between approximately 900 and 1700, focusing on aggressive intentions (contact or not), displays, pestering, and charges (Table 2). Contact aggression involves aggressive intention, leading to physical contact between the interacting individuals (e.g., hit, bite, slap, kick, trample, and wrestle), which can potentially result in injuries, therefore justifying its more “severe” status. This ethogram, previously used in both wild (*76*) and captive settings (*59*, *77*, *78*), is largely consistent with those used by Mouginot *et al.* (*45*) and Shibata and Furuichi (*44*). The only differences are our inclusion of “pest aggression” and “display” [the latter absent from Mouginot *et al.* (*45*)] and our exclusion of “nonvocal threatening” [included by Shibata and Furuichi (*44*)]. While focal follows are often the most appropriate approach in the wild, where it is rarely possible to observe an entire group simultaneously, behavioral (all-occurrence) sampling provides a valid alternative for recording rare and conspicuous events when the whole group is visible. In addition, Kaburu *et al.* (*79*) directly compared the two methods in other primate species and found no significant differences in their effectiveness, showing that both reliably capture aggressive interactions. For each aggression, both the giver and receiver were recorded, as well as whether the aggression resulted in contact between the victim and the aggressor or not. Moreover, aggression was operationalized as an event directed at an individual. This means that an individual charging three others will be counted as three separate aggressions. Likewise, a coalitionary attack on a single individual by three others will also be counted as three separate aggressions. Therefore, aggression counts reflect both the number of victims an aggressor targets (which can be multiple at once) and the number of aggressors a victim faces (which can also be multiple at once). Our approach is similar to Mouginot *et al.* (*45*) but differs from Shibata and Furuichi (*44*), who analyzed “aggression interactions” without distinguishing the numbers of aggressors or victims. Although this choice bears the risk of overestimating rates of aggression performed by coalitionary classes or received by target groups, we consider it to be the most conservative approach in keeping consistent aggression records and dynamics at both individual and dyadic levels. To ensure consistency between dyadic- and individual-level analyses, total aggression counts were adjusted accordingly, meaning that an individual aggression count corresponds to a number of victims and not a number of behaviors. Last, 693 instances of aggression targeted at individuals under 7 years of age and 3344 undirected aggressions were observed, which were all excluded from analyses to maintain a standardized way of gauging aggression among the groups and to prevent ambiguity in the direction of aggression from clouding the resultant patterns.

**Table 2.** Ethogram of all aggressive behaviors. Names and definitions are extracted from the ethogram established by Stevens *et al*. (*74*). “S” (subject) and “R” (recipient) are hypothetical subjects to contextualize the definitions.

Behavior	Description
Aggressive intention	Sudden tense hand or body movement in the direction of another individual in nonplayful contexts or hitting, kicking, etc., without locomotion. Within this category we distinguished contact from noncontact aggression
Long charge	S shows tensed running at R over more than a few meters (more than five steps)
Short charge	S shows tensed running at R over a few meters (up to five steps)
Direct display	Tensed running in the direction of parallel to or closely passing by another individual, usually while pushing an object. This can end in a collision or another contact aggression
Mutual display	Two individuals perform a directed display toward each other
Parallel display	S and R perform a display alongside each other, running in the same direction
Pest aggression	S repeatedly approaches R, throws things, swings above R, etc., with the intention to withdraw and without piloerection or play face, at times resulting in full approaches or aimed throwing of objects with piloerection

Laptops with The Observer XT version 1.14.0 and 1.17.0 (Noldus, Wageningen, The Netherlands) software installed were used to document these observations. All observers were trained on the ethogram for at least 2 weeks before starting actual observations. Interobserver reliability was tested by scoring the same video of (varied) bonobo interactions and reached at least a mean of ρ = 0.85 per observer-dyad (Spearman correlation), which is considered very strong reliability (*80*). Observers were able to recognize and distinguish all group members.

As this is an observational study, the scientific advisory board of the Royal Zoological Society of Antwerp waivered the need for ethical approval. This study conformed to the ASAB (Association for the Study of Animal Behaviour) guidelines for animal behavior research.

### Statistical analyses

All statistical analyses were performed in R (version 4.3.1; R Core Team 2025) through Rstudio (version 2023.09.01; RStudio Team 2020), and Bayesian regression models were created in the Stan computational framework and accessed using the brms package, version 2.21.0 (*81*, *82*). We used the packages emmeans (*83*), tidybayes (*84*), and bayestestR (*85*) to calculate indices of effect existence. In our regression models, we used sum-to-zero coding for all categorical predictors, because this allows one to identify the effect of each categorical predictor on the dependent variable while collapsing across the other categorical predictors, unlike treatment coding (*86*).

### Individual-level analysis

For the individual-level analyses, we fitted two Bayesian regression models with either total aggression count or contact aggression count as a dependent variable. Both models were specified with a negative binomial error distribution, which is suitable for count data (*87*). Furthermore, we specified an offset term to control for sampling effort per individual, which reflected the total observation time during which all occurrences of aggressive behaviors were scored for each individual. We included the interaction between individual sex and species and the corresponding main effects as predictors. In addition, we included adult group size (*z*-scored) and adult female proportion (*z*-scored) as control variables. Regarding the random effect structure, we included a random intercept for the group and allowed the slope for sex to vary by group (random slope). We also reran both models with a dataset consisting of only interactions between individuals >12 years (see Results).

For all models, we retained default priors for the intercept (Student’s *t* prior with 3 degrees of freedom), the variance parameters (half Student’s *t* prior with 3 degrees of freedom), and the dispersion parameter (inverse gamma prior). We ran each of the two models with three different regularizing prior specifications (*88*) for the fixed effects: a wide prior [Normal(0, 1.5)], a medium-wide prior [Normal(0, 1)], and a narrow prior [Normal(0, 0.5)]. We ran both models with four chains of 5000 iterations, 1000 of which were warm-up iterations. For all models, we checked model convergence by inspecting the trace plots, histograms of the posteriors, Gelman-Rubin diagnostics, and autocorrelation between iterations (*89*). In addition, we visually inspected the posterior predictive distribution (*90*) and the distribution of the scaled residuals through DHARMa (*91*). We identified no indications of convergence issues or model misspecification.

Throughout all analyses, we report three indices of effect existence. First is the 95% CI for the parameter estimate or the contrast, indicating the uncertainty related to the estimated value. Second is the pd, which reflects the proportion of posterior samples that is of the median’s sign. Thus, this measure can take any value between 0.5 and 1 (*85*). Third, we report BF, a measure of how well the data are explained by either the null model (no effect) or the alternative model. Depending on the direction of the effect, we report either BF01, reflecting to what extent the null model is favored over the alternative model, or BF10, reflecting the opposite. In line with recent best practices, we explicitly state here that we assumed prior odds of 1 for the null and alternative models, which means that the BF equals the posterior odds (*92*). We largely follow the classification of Lee and Wagenmakers (*93*), who classify a BF of 1 to 3 as weak evidence, a BF between 3 and 10 as moderate evidence, and a BF >10 as strong evidence. As previously mentioned, we specified each of our models with three different prior specifications. We did this because BFs are known to be very sensitive to prior specifications (*92*). We report the BFs from each of the three corresponding models in the following order: narrow prior–medium-wide prior–wide prior. For the other two indices of effect size, we report only the estimates on the basis of the models with medium-wide priors. The full model tables for all model specifications are available in the Supplementary Materials.

### Dyadic-level analysis: Social networks

We fitted Bayesian social networks through the BisonR package (*94*). For each of the 22 groups, we created one dyadic total aggression network and one dyadic contact aggression network. Compared to other methods, BISoN estimates uncertainty in the edge weights (i.e., connection between individuals) on the basis of sampling effort. More specifically, while traditional methods would give a point estimate for the edge weight in a social network, BISoN gives a distribution of edge weights for each dyad, with more variability for dyads that have been sampled at a lower frequency. This edge weight uncertainty can be modeled explicitly further downstream in the analyses.

For our case, we applied a directed count edge weight model to our data, which models the occurrence of an event per unit time. This analysis takes the direction of the interaction and the sampling effort per dyad into account. Because rates of aggression are generally quite low per unit time, we specified a relatively flat prior for the edge weights [Normal(0, 5)]. After fitting the model, we visually inspected posterior predictive distributions and a correlation plot of point and BISoN estimates. These checks did not indicate misspecification for any of the networks.

### Dyadic-level analysis: Dyadic regression

We made use of the bison_brm wrapper function that allows for running dyadic regressions on the social networks created in BisonR through brms. We specified the estimated edge weights as dependent variables in Bayesian regression models with Student’s *t* family, which is ideal for robust linear models. We included the three-way interaction between aggressor sex, recipient sex, and species and all their lower-order interactions and main effects. We added group size (*z*-scored) and sex ratio (*z*-scored) as control variables. In addition, we allowed intercepts to vary by network (group), and we controlled for individual ID by adding a multimembership random effect accounting for the presence of the same individuals across multiple dyads. We used both the raw edge weight and *z*-scored edge weight as dependent variables for both types of aggressions. When modeling raw edge weights, the analysis captures the absolute magnitude of interactions, allowing for the assessment of predictors’ effects on the original scale of the data. In contrast, *z*-scoring the edge weights standardizes them within each network, removing between-network variability in interaction scale and focusing the analysis on relative patterns of interaction within networks. Thus, we specified a total of four regression models—with total or contact aggression counts and, for each, with raw or *z*-scored edge weights. We also reran our models for a dataset consisting only of interactions of individuals >12 years old as a sensitivity analysis (see Results).

For all models, we retained default priors for the intercept (Student’s *t* prior with 3 degrees of freedom), the variance parameters (half Student’s *t* prior with 3 degrees of freedom), and Student’s *t* family’s *v* parameter (gamma prior). Similar to the individual-level analyses, we ran our models with three different regularizing prior specifications (*88*) for the fixed effects (for justification, see “Individual-level analysis”). For the raw edge weight analyses, these were a wide prior [Normal(0, 1)], a medium-wide prior [Normal(0, 0.5)], and a narrow prior [Normal(0, 0.25)]. For the *z*-scored edge weight analyses, we specified priors with lower SDs, because the distribution of the dependent variable was more compressed compared to the raw edge weight analyses. This yielded the following fixed-effect priors: a wide prior [Normal(0, 0.5)], a medium-wide prior [Normal(0, 0.25)], and a narrow prior [Normal(0, 0.1)].

To include the uncertainty of the edge weights, we fitted 50 submodels to 50 datasets that were uniquely sampled from the edge weight distributions. We ran each submodel with two chains of 3000 iterations, 1000 of which were warm-up samples. Thus, this yielded 200,000 posterior samples per regression model. For all models, we checked model convergence by inspecting the trace plots, histograms of the posteriors, Gelman-Rubin diagnostics within each of the submodels, and autocorrelation between iterations for the first chain (*89*). In addition, we visually inspected the posterior predictive distribution (*90*) and the distribution of the scaled residuals through DHARMa (*91*). We identified no indications of convergence issues or model misspecification.
