# Supplementary material for: Chimpanzees are not more aggressive than bonobos but target sexes differently
Source: Sci Adv. 2026 Mar 11;12(11):eadz2433. doi: 10.1126/sciadv.adz2433 (PMC12978231; doi:10.1126/sciadv.adz2433)
Supplement: Supplementary file 1 — Tables S1 to S24 [file sciadv.adz2433_sm.pdf]

Science Advances
AAAS

Supplementary Materials for

Chimpanzees are not more aggressive than bonobos but target sexes differently

Emile Bryon *et al.*

Corresponding author: Emile Bryon, e.bryon@uu.nl; Edwin J. C. van Leeuwen, e.j.c.vanleeuwen@uu.nl; Nicky Staes, nickystaes2@gmail.com

Sci. Adv. **12**, eadz2433 (2026)
DOI: 10.1126/sciadv.adz2433

This PDF file includes:

Tables S1 to S24

Individual level analyses

Supplementary Table 1 – Model tables for the individual-level total aggression analyses. Note that all categorical predictors were sum-coded, with Female and Bonobo coded as 1.

	**Narrow prior**		**Medium-wide prior**		**Wide prior**	
Predictors	*Incidence Rate Ratios*	*CI (95%)*	*Incidence Rate Ratios*	*CI (95%)*	*Incidence Rate Ratios*	*CI (95%)*
Intercept	0.11	0.07 – 0.15	0.11	0.07 – 0.16	0.11	0.07 – 0.16
female proportion (z-scored)	0.84	0.60 – 1.19	0.83	0.57 – 1.20	0.83	0.57 – 1.21
group size (z-scored)	0.92	0.68 – 1.29	0.92	0.65 – 1.32	0.92	0.64 – 1.34
sex1	0.63	0.51 – 0.82	0.62	0.49 – 0.80	0.62	0.49 – 0.80
species1	1.06	0.73 – 1.50	1.05	0.70 – 1.54	1.05	0.70 – 1.54
sex1:species1	1.46	1.15 – 1.82	1.48	1.17 – 1.88	1.49	1.18 – 1.89
**Random Effects**						
τ_{00}	0.70 $_{group}$		0.71 $_{group}$		0.72 $_{group}$	
τ_{11}	0.33 $_{group.sex1}$		0.34 $_{group.sex1}$		0.33 $_{group.sex1}$	
N	22 $_{group}$		22 $_{group}$		22 $_{group}$	
Observations	189		189		189	

Supplementary Table 2 – Model tables for the individual-level total aggression analyses including only interactions between >12 year old individuals. Note that all categorical predictors were sum-coded, with Female and Bonobo coded as 1.

	**Narrow prior**		**Medium-wide prior**		**Wide prior**	
Predictors	*Incidence Rate Ratios*	*CI (95%)*	*Incidence Rate Ratios*	*CI (95%)*	*Incidence Rate Ratios*	*CI (95%)*
Intercept	0.06	0.03 – 0.09	0.06	0.03 – 0.10	0.06	0.03 – 0.10
female proportion (z-scored)	0.70	0.44 – 1.13	0.65	0.38 – 1.13	0.64	0.36 – 1.10
group size (z-scored)	1.02	0.66 – 1.64	0.99	0.60 – 1.69	0.98	0.58 – 1.71
sex1	0.59	0.44 – 0.78	0.57	0.42 – 0.78	0.57	0.42 – 0.77
species1	0.77	0.47 – 1.24	0.72	0.41 – 1.24	0.70	0.38 – 1.23
sex1:species1	1.32	0.97 – 1.75	1.34	0.99 – 1.80	1.35	0.99 – 1.82
**Random Effects**						
τ_{00}	1.05 group		1.07 group		1.08 group	
τ_{11}	0.46 group.sex1		0.45 group.sex1		0.45 group.sex1	
N	22 group		22 group		22 group	
Observations	162		162		162	

Supplementary Table 3 – Model tables for the individual-level contact aggression analyses. Note that all categorical predictors were sum-coded, with Female and Bonobo coded as 1.

	**Narrow prior**		**Medium-wide prior**		**Wide prior**	
Predictors	*Incidence Rate Ratios*	*CI (95%)*	*Incidence Rate Ratios*	*CI (95%)*	*Incidence Rate Ratios*	*CI (95%)*
Intercept	0.04	0.02 – 0.06	0.04	0.02 – 0.06	0.04	0.02 – 0.06
female proportion (z-scored)	0.97	0.65 – 1.43	0.97	0.64 – 1.47	0.97	0.63 – 1.49
group size (z-scored)	0.97	0.67 – 1.43	0.97	0.64 – 1.48	0.97	0.65 – 1.50
sex1	0.61	0.47 – 0.83	0.59	0.45 – 0.81	0.59	0.44 – 0.80
species1	1.02	0.68 – 1.52	1.02	0.65 – 1.59	1.02	0.64 – 1.58
sex1:species1	1.31	0.99 – 1.71	1.34	0.99 – 1.78	1.34	1.00 – 1.79
**Random Effects**						
τ_{00}	0.83 $_{group}$		0.84 $_{group}$		0.84 $_{group}$	
τ_{11}	0.46 $_{group.sex1}$		0.46 $_{group.sex1}$		0.46 $_{group.sex1}$	
N	22 $_{group}$		22 $_{group}$		22 $_{group}$	
Observations	189		189		189	

Supplementary Table 4 – Model tables for the individual-level contact aggression analyses including only interactions between >12 year old individuals. Note that all categorical predictors were sum-coded, with Female and Bonobo coded as 1.

	**Narrow prior**		**Medium-wide prior**		**Wide prior**	
Predictors	*Incidence Rate Ratios*	*CI (95%)*	*Incidence Rate Ratios*	*CI (95%)*	*Incidence Rate Ratios*	*CI (95%)*
Intercept	0.02	0.01 – 0.03	0.02	0.01 – 0.03	0.02	0.01 – 0.03
female proportion (z-scored)	0.77	0.45 – 1.30	0.72	0.38 – 1.29	0.71	0.36 – 1.30
group size (z-scored)	1.09	0.69 – 1.81	1.07	0.62 – 1.96	1.07	0.59 – 1.99
sex1	0.59	0.41 – 0.86	0.56	0.38 – 0.83	0.55	0.37 – 0.82
species1	0.70	0.41 – 1.16	0.63	0.33 – 1.14	0.61	0.31 – 1.14
sex1:species1	1.25	0.86 – 1.77	1.28	0.87 – 1.87	1.30	0.87 – 1.90
**Random Effects**						
τ_{00}	1.12 $_{group}$		1.16 $_{group}$		1.19 $_{group}$	
τ_{11}	0.60 $_{group.sex1}$		0.59 $_{group.sex1}$		0.59 $_{group.sex1}$	
N	22 $_{group}$		22 $_{group}$		22 $_{group}$	
Observations	162		162		162	

Dyadic level analyses (raw edge weights)

Supplementary Table 5 – Model tables for the dyadic total aggression analyses (raw edge weights). Note that all categorical predictors were sum-coded, with Female and Bonobo coded as 1.

	**Narrow prior**		**Medium-wide prior**		**Wide prior**	
Predictors	*Estimates*	*CI (95%)*	*Estimates*	*CI (95%)*	*Estimates*	*CI (95%)*
Intercept	-5.42	-5.85 – -4.99	-5.36	-5.81 – -4.92	-5.36	-5.80 – -4.91
female proportion (z-scored)	0.00	-0.33 – 0.33	0.03	-0.38 – 0.44	0.03	-0.42 – 0.48
group size (z-scored)	-0.23	-0.53 – 0.08	-0.31	-0.69 – 0.07	-0.34	-0.76 – 0.08
aggressor.sex1	-0.45	-0.60 – -0.30	-0.48	-0.65 – -0.31	-0.51	-0.68 – -0.34
recipient.sex1	-0.32	-0.48 – -0.17	-0.37	-0.53 – -0.21	-0.39	-0.56 – -0.23
Species1	0.10	-0.22 – 0.43	0.13	-0.27 – 0.54	0.14	-0.31 – 0.60
aggressor.sex1:recipient.sex1	-0.06	-0.20 – 0.08	-0.06	-0.21 – 0.08	-0.05	-0.20 – 0.11
aggressor.sex1:Species1	0.29	0.14 – 0.45	0.29	0.12 – 0.45	0.28	0.12 – 0.45
recipient.sex1:Species1	-0.25	-0.41 – -0.09	-0.27	-0.44 – -0.11	-0.28	-0.44 – -0.12
aggressor.sex1:recipient.sex1:Species1	-0.08	-0.21 – 0.06	-0.07	-0.21 – 0.08	-0.07	-0.22 – 0.08
**Random Effects**						
σ^2	2.02		2.02		2.00	
τ_{00}	0.77_{group}		0.78_{group}		0.80_{group}	
τ_{00}	$0.99_{mm(Aggr, Rec)}$		$0.99_{mm(Aggr, Rec)}$		$0.99_{mm(Aggr, Rec)}$	

N	22_{group}	22_{group}	22_{group}
N	$187_{individuals}$	$187_{individuals}$	$187_{individuals}$
Observations	2102	2102	2102

Supplementary Table 6 – Within-species contrasts for the raw edge weight analysis of dyadic total aggression count.

Contrast		Δ	95% CI	*pd*	BF
			Bonobos		
F-F	M-F	-0.64	-1.31 – 0.01	0.973	BF_{10}: 1.71 – 1.11 – 0.52
F-F	F-M	-1.56	-2.18 – -0.95	1.000	BF_{10}: >100 – >100 – >100
F-F	M-M	-1.68	-2.57 – -0.79	0.999	BF_{10}: >100 – >100 – >100
M-F	F-M	-0.91	-1.49 – -0.32	0.999	BF_{10}: 21.26 – 14.07 – 5.40
M-F	M-M	-1.03	-1.85 – -0.25	0.995	BF_{10}: 5.54 – 5.62 – 2.90
F-M	M-M	-0.13	-0.91 – 0.68	0.627	BF_{01}: 2.72 – 4.70 – 8.33
			Chimpanzees		
F-F	M-F	-1.53	-1.97 – -1.10	1.000	BF_{10}: >100 – >100 – >100
F-F	F-M	-0.19	-0.66 – 0.31	0.781	BF_{01}: 3.96 – 5.84 – 12.66
F-F	M-M	-1.73	-2.39 – -1.11	1.000	BF_{10}: >100 – >100 – >100
M-F	F-M	1.34	0.91 – 1.78	1.000	BF_{10}: >100 – >100 – >100
M-F	M-M	-0.20	-0.71 – 0.29	0.792	BF_{01}: 3.12 – 5.77 – 9.23
F-M	M-M	-1.56	-2.10 – -1.01	1.000	BF_{10}: >100 – >100 – >100

Supplementary Table 7 – Model tables for the dyadic <u>total</u> aggression analyses (raw edge weights) including only interactions between >12 year old individuals. Note that all categorical predictors were sum-coded, with Female and Bonobo coded as 1.

	**Narrow prior**		**Medium-wide prior**		**Wide prior**	
Predictors	*Estimates*	*CI (95%)*	*Estimates*	*CI (95%)*	*Estimates*	*CI (95%)*
Intercept	-5.59	-6.07 – -5.12	-5.54	-6.03 – -5.05	-5.52	-6.03 – -5.00
female proportion (z-scored)	-0.06	-0.42 – 0.30	-0.07	-0.54 – 0.41	-0.10	-0.62 – 0.43
group size (z-scored)	-0.18	-0.49 – 0.14	-0.25	-0.65 – 0.15	-0.29	-0.75 – 0.17
aggressor.sex1	-0.47	-0.66 – -0.29	-0.52	-0.72 – -0.32	-0.53	-0.74 – -0.32
recipient.sex1	-0.32	-0.51 – -0.13	-0.37	-0.57 – -0.16	-0.37	-0.59 – -0.14
Species1	0.04	-0.30 – 0.38	0.03	-0.42 – 0.48	0.04	-0.47 – 0.53
aggressor.sex1:recipient.sex1	-0.06	-0.23 – 0.10	-0.05	-0.23 – 0.13	-0.05	-0.24 – 0.14
aggressor.sex1:Species1	0.25	0.07 – 0.44	0.25	0.04 – 0.45	0.23	0.02 – 0.43
recipient.sex1:Species1	-0.22	-0.41 – -0.03	-0.24	-0.44 – -0.02	-0.25	-0.46 – -0.03
aggressor.sex1:recipient.sex1:Species1	-0.09	-0.26 – 0.08	-0.07	-0.25 – 0.10	-0.06	-0.24 – 0.12
**Random Effects**						
σ^2	2.00		1.99		1.98	
τ_{00}	0.77_{group}		0.80_{group}		0.83_{group}	
τ_{00}	$1.00_{mm(Aggr, Rec)}$		$0.99_{mm(Aggr, Rec)}$		$1.00_{mm(Aggr, Rec)}$	
N	22_{group}		22_{group}		22_{group}	

N	160 individuals	160 individuals	160 individuals
Observations	1666	1666	1666

Supplementary Table 8 – Species comparisons for the different dyad compositions for the raw edge weight analysis of dyadic total aggression count including only interactions between >12 year old individuals.

**Contrast**	**Δ**	**95% CI**	***pd***	**BF**
F-F	-0.078	-1.099 – 0.965	0.560	BF01: 2.21 – 3.84 – 6.80
M-F	-0.764	-1.825 – 0.323	0.918	BF01: 0.69 – 1.36 – 2.90
F-M	1.180	0.129 – 2.217	0.985	BF10: 16.54 – 3.01 – 1.08
M-M	-0.098	-1.443 – 1.204	0.558	BF01: 1.75 – 2.94 – 5.56

Supplementary Table 9 – Within-species contrasts for the raw edge weight analysis of dyadic total aggression count including only interactions between >12 year old individuals.

Contrast		Δ	95% CI	*pd*	BF
			Bonobos		
F-F	M-F	-0.80	-1.65 – 0.04	0.970	BF_{10}: 2.55 – 1.20 – 0.66
F-F	F-M	-1.48	-2.33 – -0.64	0.998	BF_{10}: >100 – 18.50 – 23.74
F-F	M-M	-1.77	-2.93 – -0.61	0.998	BF_{10}: >100 – >100 – >100
M-F	F-M	-0.67	-1.48 – 0.16	0.941	BF_{10}: 1.42 – 0.77 – 0.32
M-F	M-M	-0.97	-2.02 – 0.13	0.960	BF_{10}: 1.67 – 1.23 – 0.65
F-M	M-M	-0.30	-1.33 – 0.71	0.716	BF_{01}: 1.94 – 3.29 – 5.78
			Chimpanzees		
F-F	M-F	-1.49	-1.94 – -1.01	1.000	BF_{10}: >100 – >100 – >100
F-F	F-M	-0.21	-0.66 – 0.24	0.820	BF_{01}: 3.29 – 5.69 – 12.04
F-F	M-M	-1.78	-2.46 – -1.11	1.000	BF_{10}: >100 – >100 – >100
M-F	F-M	1.27	0.84 – 1.70	1.000	BF_{10}: >100 – >100 – >100
M-F	M-M	-0.30	-0.85 – 0.23	0.867	BF_{01}: 2.36 – 4.04 – 9.06
F-M	M-M	-1.58	-2.13 – -1.04	1.000	BF_{10}: >100 – >100 – >100

Supplementary Table 10 – Model tables for the dyadic contact aggression analyses (raw edge weights). Note that all categorical predictors were sum-coded, with Female and Bonobo coded as 1.

	**Narrow prior**		**Medium-wide prior**		**Wide prior**	
Predictors	*Estimates*	*CI (95%)*	*Estimates*	*CI (95%)*	*Estimates*	*CI (95%)*
Intercept	-6.29	-6.65 – -5.92	-6.28	-6.67 – -5.90	-6.27	-6.66 – -5.88
female proportion (z-scored)	0.04	-0.25 – 0.35	0.05	-0.30 – 0.41	0.06	-0.33 – 0.45
group size (z-scored)	-0.16	-0.42 – 0.12	-0.19	-0.51 – 0.14	-0.21	-0.57 – 0.15
aggressor.sex1	-0.28	-0.43 – -0.12	-0.29	-0.46 – -0.13	-0.29	-0.46 – -0.13
recipient.sex1	-0.20	-0.35 – -0.05	-0.22	-0.39 – -0.05	-0.23	-0.40 – -0.05
Species1	0.11	-0.19 – 0.40	0.14	-0.22 – 0.50	0.14	-0.25 – 0.53
aggressor.sex1:recipient.sex1	0.02	-0.13 – 0.17	0.01	-0.14, 0.17	0.03	-0.14 – 0.19
aggressor.sex1:Species1	0.09	-0.06 – 0.24	0.08	-0.07, 0.24	0.09	-0.07 – 0.26
recipient.sex1:Species1	-0.15	-0.30 – -0.00	-0.16	-0.33, 0.01	-0.15	-0.32, 0.00

aggressor.sex1:recipient.sex1:Species1	-0.02	-0.17 – 0.14	-0.03	-0.19, 0.13	-0.02	-0.17, 0.13
**Random Effects**						
σ^2	2.13		2.13		2.15	
τ_{00}	0.62 $_{group}$		0.64 $_{group}$		0.66 $_{group}$	
τ_{00}	0.58 $_{mm(Aggr, Rec)}$		0.56 $_{mm(Aggr, Rec)}$		0.57 $_{mm(Aggr, Rec)}$	
N	22 $_{group}$		22 $_{group}$		22 $_{group}$	
N	187 $_{individuals}$		187 $_{individuals}$		187 $_{individuals}$	
Observations	2102		2102		2102	

Supplementary Table 11 – Within-species contrasts for the raw edge weight analysis of dyadic contact aggression count.

Contrast		Δ	95% CI	*pd*	BF
			Bonobos		
F-F	M-F	-0.45	-1.08 – 0.19	0.918	BF_{01}: 1.49 – 2.46 – 6.71
F-F	F-M	-0.79	-1.45 – -0.10	0.985	BF_{10}: 5.15 – 1.94 – 1.01
F-F	M-M	-1.19	-2.04 – -0.33	0.997	BF_{10}: 17.42 – 8.46 – 4.02
M-F	F-M	-0.34	-1.03 – 0.35	0.832	BF_{01}: 1.91 – 3.55 – 6.77
M-F	M-M	-0.73	-1.60 – 0.12	0.955	BF_{01}: 0.54 – 1.13 – 1.95
F-M	M-M	-0.40	-1.25 – 0.44	0.823	BF_{01}: 1.62 – 3.08 – 5.72
			Chimpanzees		
F-F	M-F	-0.67	-1.13 – -0.19	0.997	BF_{10}: 30.42 – 4.77 – 6.63
F-F	F-M	-0.05	-0.49 – 0.43	0.581	BF_{01}: 4.40 – 8.24 – 16.50
F-F	M-M	-0.87	-1.46 – -0.31	0.999	BF_{10}: 46.02 – 14.92 – 7.46
M-F	F-M	0.63	0.17 – 1.05	0.998	BF_{10}: 17.20 – 5.39 – 2.58
M-F	M-M	-0.22	-0.74 – 0.33	0.778	BF_{01}: 3.19 – 5.18 – 10.59
F-M	M-M	-0.84	-1.36 – -0.31	0.999	BF_{10}: 19.26 – 20.33 – 5.56

Supplementary Table 12 – Model tables for the dyadic contact aggression analyses (raw edge weights) including only interactions between >12 year old individuals. Note that all categorical predictors were sum-coded, with Female and Bonobo coded as 1.

	**Narrow prior**		**Medium-wide prior**		**Wide prior**	
Predictors	*Estimates*	*CI (95%)*	*Estimates*	*CI (95%)*	*Estimates*	*CI (95%)*
Intercept	-6.48	-6.89 – -6.06	-6.44	-6.89 – -5.99	-6.45	-6.91 – -6.00
female proportion (z-scored)	-0.06	-0.39 – 0.28	-0.06	-0.49 – 0.37	-0.07	-0.55 – 0.42
group size (z-scored)	-0.12	-0.40 – 0.18	-0.17	-0.53 – 0.20	-0.18	-0.58 – 0.22
aggressor.sex1	-0.28	-0.47 – -0.08	-0.28	-0.51 – -0.05	-0.29	-0.52 – -0.04
recipient.sex1	-0.15	-0.33 – 0.04	-0.19	-0.40 – 0.02	-0.17	-0.38 – 0.04
Species1	0.01	-0.31 – 0.33	0.01	-0.40 – 0.42	-0.01	-0.47 – 0.45
aggressor.sex1:recipient.sex1	0.04	-0.17 – 0.23	0.03	-0.18 – 0.23	0.04	-0.15 – 0.24
aggressor.sex1:Species1	0.07	-0.11 – 0.25	0.08	-0.13 – 0.29	0.08	-0.14 – 0.34
recipient.sex1:Species1	-0.08	-0.27 – 0.13	-0.10	-0.31 – 0.10	-0.12	-0.33 – 0.09

aggressor.sex1:recipient.sex1:Species1	-0.02	-0.22 – 0.17	-0.02	-0.22 – 0.19	-0.01	-0.21 – 0.19
**Random Effects**						
σ^2	2.11		2.13		2.13	
τ_{00}	0.68 group		0.71 group		0.72 group	
τ_{00}	0.46 mm(Aggr, Rec)		0.49 mm(Aggr, Rec)		0.46 mm(Aggr, Rec)	
N	22 group		22 group		22 group	
N	160 individuals		160 individuals		160 individuals	
Observations	1666		1666		1666	

Supplementary Table 13 – Species comparisons for the different dyad compositions for the raw edge weight analysis of dyadic contact aggression count including only interactions between >12 year old individuals.

**Contrast**	**Δ**	**95% CI**	***pd***	**BF**
F-F	-0.063	-1.029 – 0.918	0.552	BF01: 2.42 – 4.16 – 7.68
M-F	-0.296	-1.320 – 0.694	0.722	BF01: 1.98 – 3.44 – 5.67
F-M	0.438	-0.631 – 1.459	0.797	BF01: 1.63 – 2.63 – 5.48
M-M	0.036	-1.297 – 1.337	0.522	BF01: 1.81 – 2.96 – 5.52

Supplementary Table 14 – Within-species contrasts for the raw edge weight analysis of dyadic contact aggression count including only interactions between >12 year old individuals.

Contrast		Δ	95% CI	*pd*	BF
			Bonobos		
F-F	M-F	-0.39	-1.28 – 0.48	0.812	BF_{01}: 1.57 – 3.06 – 6.33
F-F	F-M	-0.57	-1.48 – 0.30	0.899	BF_{01}: 1.46 – 2.07 – 4.89
F-F	M-M	-1.00	-2.21 – 0.23	0.941	BF_{10}: 1.92 – 1.09 – 0.54
M-F	F-M	-0.19	-1.09 – 0.73	0.659	BF_{01}: 2.42 – 3.98 – 7.17
M-F	M-M	-0.60	-1.79 – 0.56	0.844	BF_{01}: 1.21 – 1.98 – 3.71
F-M	M-M	-0.42	-1.68 – 0.82	0.745	BF_{01}: 1.24 – 2.52 – 4.46
			Chimpanzees		
F-F	M-F	-0.62	-1.12 – -0.13	0.995	BF_{10}: 5.62 – 3.25 – 2.95
F-F	F-M	-0.08	-0.57 – 0.40	0.652	BF_{01}: 4.20 – 7.45 – 16.91
F-F	M-M	-0.90	-1.49 – -0.29	0.999	BF_{10}: 12.47 – 14.47 – 3.95
M-F	F-M	0.54	0.03 – 1.08	0.978	BF_{10}: 4.59 – 1.13 – 2.35
M-F	M-M	-0.27	-0.84 – 0.30	0.819	BF_{01}: 2.25 – 4.59 – 10.68
F-M	M-M	-0.82	-1.37 – -0.25	0.998	BF_{10}: 21.59 – 7.12 – 5.28

Dyadic level analyses (z-scored edge weights)

Supplementary Table 15 – Model tables for the dyadic total aggression analyses (z-scored edge weights). Note that all categorical predictors were sum-coded, with Female and Bonobo coded as 1.

	**Narrow prior**		**Medium-wide prior**		**Wide prior**	
Predictors	*Estimates*	*CI (95%)*	*Estimates*	*CI (95%)*	*Estimates*	*CI (95%)*
Intercept	0.16	0.08 – 0.24	0.17	0.09 – 0.25	0.16	0.08 – 0.25
aggressor.sex1	-0.16	-0.22 – -0.10	-0.17	-0.23 – -0.11	-0.18	-0.24 – -0.12
recipient.sex1	-0.11	-0.17 – -0.06	-0.13	-0.19 – -0.07	-0.13	-0.18 – -0.07
Species1	0.00	-0.07 – 0.07	0.01	-0.07 – 0.04	0.01	-0.07 – 0.08
aggressor.sex1:recipient.sex1	-0.02	-0.07 – 0.03	-0.02	-0.07 – 0.08	-0.02	-0.07 – 0.04
aggressor.sex1:Species1	0.11	0.06 – 0.17	0.12	0.06 – 0.17	0.12	0.06 – 0.18
recipient.sex1:Species1	-0.10	-0.15 – -0.04	-0.10	-0.16 – -0.04	-0.10	-0.16 – -0.04
aggressor.sex1:recipient.sex1:Species1	-0.02	-0.08 – 0.03	-0.02	-0.08 – 0.03	-0.03	-0.08 – 0.03
**Random Effects**						
σ^2	0.79		0.79		0.79	
τ_{00}	0.03 $_{group}$		0.04 $_{group}$		0.04 $_{group}$	

τ_{00}	$0.31_{mm(Aggr, Rec)}$	$0.32_{mm(Aggr, Rec)}$	$0.32_{mm(Aggr, Rec)}$
N	22_{group}	22_{group}	22_{group}
N	$187_{individuals}$	$187_{individuals}$	$187_{individuals}$
Observations	2102	2102	2102

Supplementary Table 16 – Within-species contrasts for the z-scored edge weight analysis of dyadic total aggression count.

Contrast		Δ	95% CI	*pd*	BF
			Bonobos		
F-F	M-F	-0.20	-0.45 – 0.06	0.936	BF_{01}: 0.97 – 2.32 – 3.59
F-F	F-M	-0.54	-0.77 – -0.30	1.000	BF_{10}: >100 – >100 – >100
F-F	M-M	-0.57	-0.88 – -0.27	0.999	BF_{10}: 90.78 – 115.53 – 39.21
M-F	F-M	-0.34	-0.57 – -0.10	0.998	BF_{10}: 17.63 – 6.54 – 3.99
M-F	M-M	-0.37	-0.69 – -0.07	0.992	BF_{10}: 4.68 – 2.69 – 1.44
F-M	M-M	-0.04	-0.34 – 0.27	0.596	BF_{01}: 2.80 – 6.26 – 12.76
			Chimpanzees		
F-F	M-F	-0.56	-0.73 – -0.40	1.000	BF_{10}: >100 – >100 – >100
F-F	F-M	-0.04	-0.21 – 0.14	0.654	BF_{01}: 4.08 – 10.59 – 21.24
F-F	M-M	-0.63	-0.85 – -0.41	1.000	BF_{10}: >100 – >100 – >100
M-F	F-M	0.53	0.38 – 0.68	1.000	BF_{10}: >100 – >100 – >100
M-F	M-M	-0.07	-0.25 – 0.12	0.77	BF_{01}: 4.05 – 8.39 – 17.69
F-M	M-M	-0.60	-0.79 – -0.41	1.000	BF_{10}: >100 – >100 – >100

Supplementary Table 17 – Model tables for the dyadic total aggression analyses (z-scored edge weights) including only interactions between >12 year old individuals. Note that all categorical predictors were sum-coded, with Female and Bonobo coded as 1.

	**Narrow prior**		**Medium-wide prior**		**Wide prior**	
Predictors	*Estimates*	*CI (95%)*	*Estimates*	*CI (95%)*	*Estimates*	*CI (95%)*
Intercept	0.16	0.07 – 0.25	0.17	0.07 – 0.27	0.17	0.07 – 0.27
aggressor.sex1	-0.16	-0.23 – -0.09	-0.17	-0.26 – -0.11	-0.18	-0.26 – -0.11
recipient.sex1	-0.10	-0.17 – -0.04	-0.12	-0.20 – -0.05	-0.12	-0.20 – -0.04
Species1	0.00	-0.07 – 0.08	0.01	-0.08 – 0.09	0.01	-0.08 – 0.10
aggressor.sex1:recipient.sex1	-0.02	-0.09 – 0.05	-0.02	-0.09 – 0.05	-0.02	-0.09 – 0.05
aggressor.sex1:Species1	0.10	0.03 – 0.17	0.10	0.02 – 0.17	0.10	0.02 – 0.18
recipient.sex1:Species1	-0.09	-0.15 – -0.02	-0.10	-0.18 – -0.03	-0.10	-0.17 – -0.02
aggressor.sex1:recipient.sex1:Species1	-0.03	-0.09 – 0.04	-0.02	-0.10 – 0.05	-0.03	-0.10 – 0.04
**Random Effects**						
σ^2	0.79		0.80		0.80	
τ_{00}	0.04 $_{group}$		0.04 $_{group}$		0.04 $_{group}$	
τ_{00}	0.29 $_{mm(Aggr, Rec)}$		0.29 $_{mm(Aggr, Rec)}$		0.29 $_{mm(Aggr, Rec)}$	
N	22 $_{group}$		22 $_{group}$		22 $_{group}$	

N	160 individuals	160 individuals	160 individuals
Observations	1666	1666	1666

Supplementary Table 18 – Species comparisons for the different dyad compositions for the z-scored edge weight analysis of dyadic total aggression count including only interactions between >12 year old individuals.

**Contrast**	**Δ**	**95% CI**	***pd***	**BF**
F-F	-0.037	-0.270 – 0.200	0.622	BF01: 3.40 – 7.94 – 15.57
M-F	-0.325	-0.605 – -0.046	0.990	BF10: 5.03 – 1.93 – 0.84
F-M	0.465	0.177 – 0.747	0.999	BF10: 56.26 – 16.97 – 11.96
M-M	-0.014	-0.421 – 0.393	0.527	BF01: 2.02 – 4.80 – 9.35

Supplementary Table 19 – Within-species contrasts for the z-scored edge weight analysis of dyadic <u>total</u> aggression count including only interactions between >12 year old individuals.

Contrast		Δ	95% CI	*pd*	BF
			Bonobos		
F-F	M-F	-0.26	-0.56 – 0.04	0.955	BF_{01}: 0.86 – 1.53 – 3.11
F-F	F-M	-0.54	-0.83 – -0.23	0.999	BF_{10}: 59.86 – 43.33 – 15.99
F-F	M-M	-0.62	-1.02 – -0.22	0.999	BF_{10}: 16.82 – 19.08 – 3.91
M-F	F-M	-0.28	-0.60 – 0.04	0.956	BF_{10}: 1.47 – 0.68 – 0.39
M-F	M-M	-0.37	-0.79 – 0.05	0.957	BF_{10}: 1.42 – 0.92 – 0.39
F-M	M-M	-0.09	-0.49 – 0.32	0.670	BF_{01}: 2.14 – 4.31 – 8.89
			Chimpanzees		
F-F	M-F	-0.55	-0.72 – -0.37	1.000	BF_{10}: >100 – >100 – >100
F-F	F-M	-0.03	-0.23 – 0.15	0.637	BF_{01}: 3.96 – 10.21 – 20.40
F-F	M-M	-0.60	-0.84 – -0.38	1.000	BF_{10}: >100 – >100 – >100
M-F	F-M	0.51	0.33 – 0.69	1.000	BF_{10}: >100 – >100 – >100
M-F	M-M	-0.06	-0.26 – 0.14	0.713	BF_{01}: 3.44 – 8.42 – 15.59
F-M	M-M	-0.57	-0.79 – -0.34	1.000	BF_{10}: >100 – >100 – >100

Supplementary Table 20 – Model tables for the dyadic contact aggression analyses (z-scored edge weights). Note that all categorical predictors were sum-coded, with Female and Bonobo coded as 1.

	**Narrow prior**		**Medium-wide prior**		**Wide prior**	
Predictors	*Estimates*	*CI (95%)*	*Estimates*	*CI (95%)*	*Estimates*	*CI (95%)*
Intercept	0.11	0.04 – 0.18	0.11	0.04 – 0.18	0.11	0.04 – 0.18
aggressor.sex1	-0.10	-0.16 – -0.04	-0.10	-0.16 – -0.04	-0.10	-0.16 – -0.04
recipient.sex1	-0.07	-0.13 – -0.01	-0.08	-0.14 – -0.02	-0.08	-0.14 – -0.02
Species1	0.01	-0.05 – 0.06	0.01	-0.05 – 0.07	0.01	-0.05 – 0.07
aggressor.sex1:recipient.sex1	0.01	-0.05, 0.06	0.01	-0.05 – 0.07	0.00	-0.06 – 0.07
aggressor.sex1:Species1	0.04	-0.01, 0.10	0.04	-0.02 – 0.10	0.04	-0.02, 0.10
recipient.sex1:Species1	-0.05	-0.11, 0.00	-0.06	-0.12 – -0.00	-0.06	-0.12 – 0.00
aggressor.sex1:recipient.sex1:Species1	-0.01	-0.06, 0.05	-0.01	-0.07 – 0.05	-0.01	-0.07 – 0.05
**Random Effects**						
σ^2	0.86		0.86		0.86	
τ_{00}	0.03 $_{group}$		0.03 $_{group}$		0.03 $_{group}$	

τ_{00}	0.14 $_{mm(Aggr, Rec)}$	0.17 $_{mm(Aggr, Rec)}$	0.15 $_{mm(Aggr, Rec)}$
N	22 $_{group}$	22 $_{group}$	22 $_{group}$
N	187 $_{individuals}$	187 $_{individuals}$	187 $_{individuals}$
Observations	2102	2102	2102

Supplementary Table 21 – Within-species contrasts for the z-scored edge weight analysis of dyadic contact aggression count.

**Contrast**		**Δ**	**95% CI**	***pd***	**BF**
			Bonobos		
F-F	M-F	-0.13	-0.37 – 0.12	0.842	BF_{01}: 2.23 – 4.70 – 9.86
F-F	F-M	-0.28	-0.53 – -0.05	0.990	BF_{01}: 0.33 – 0.53 – 1.30
F-F	M-M	-0.39	-0.69 – -0.08	0.994	BF_{10}: 8.64 – 3.81 – 1.58
M-F	F-M	-0.15	-0.42 – 0.10	0.880	BF_{01}: 1.80 – 3.93 – 6.94
M-F	M-M	-0.27	-0.58 – 0.04	0.951	BF_{01}: 0.72 – 1.52 – 3.41
F-M	M-M	-0.11	-0.41 – 0.21	0.752	BF_{01}: 1.94 – 4.93 – 9.33
			Chimpanzees		
F-F	M-F	-0.25	-0.42 – -0.08	0.998	BF_{10}: 34.66 – 5.81 – 2.68
F-F	F-M	0.00	-0.17 – 0.18	0.515	BF_{01}: 4.30 – 11.77 – 21.60
F-F	M-M	-0.32	-0.53 – -0.12	0.999	BF_{10}: 36.64 – 11.50 – 3.90
M-F	F-M	0.26	0.09 – 0.43	0.999	BF_{10}: 10.58 – 8.97 – 1.36
M-F	M-M	-0.07	-0.27 – 0.13	0.747	BF_{01}: 3.61 – 7.66 – 14.99
F-M	M-M	-0.33	-0.53 – -0.12	0.999	BF_{10}: 33.99 – 18.23 – 2.04

Supplementary Table 22 – Model tables for the dyadic contact aggression analyses (z-scored edge weights) including only interactions between >12 year old individuals. Note that all categorical predictors were sum-coded, with Female and Bonobo coded as 1.

	Narrow prior		Medium-wide prior		Wide prior	
Predictors	*Estimates*	*CI (95%)*	*Estimates*	*CI (95%)*	*Estimates*	*CI (95%)*
Intercept	0.11	0.02 – 0.19	0.11	0.02 – 0.20	0.11	0.03 – 0.20
aggressor.sex1	-0.10	-0.17 – -0.02	-0.11	-0.19 – -0.03	-0.10	-0.18 – -0.03
recipient.sex1	-0.05	-0.13 – 0.02	-0.06	-0.15 – 0.02	-0.06	-0.15 – 0.02
Species1	-0.00	-0.07 – 0.07	0.00	-0.07 – 0.08	0.01	-0.07 – 0.08
aggressor.sex1:recipient.sex1	0.01	-0.06 – 0.08	0.01	-0.07 – 0.08	0.02	-0.06 – 0.09
aggressor.sex1:Species1	0.04	-0.04 – 0.11	0.03	-0.05 – 0.12	0.03	-0.05 – 0.11
recipient.sex1:Species1	-0.03	-0.10 – 0.05	-0.04	-0.11 – 0.04	-0.04	-0.13 – 0.05
aggressor.sex1:recipient.sex1:Species1	-0.01	-0.08 – 0.07	-0.02	-0.10 – 0.07	-0.00	-0.08 – 0.08
**Random Effects**						
σ^2	0.87		0.87		0.87	
τ_{00}	0.03 $_{group}$		0.03 $_{group}$		0.03 $_{group}$	

τ_{00}	0.12 $_{mm(Aggr, Rec)}$	0.11 $_{mm(Aggr, Rec)}$	0.13 $_{mm(Aggr, Rec)}$
N	22 $_{group}$	22 $_{group}$	22 $_{group}$
N	160 $_{individuals}$	160 $_{individuals}$	160 $_{individuals}$
Observations	1666	1666	1666

Supplementary Table 23 – Species comparisons for the different dyad compositions for the z-scored edge weight analysis of dyadic contact aggression count including only interactions between >12 year old individuals.

Contrast	Δ	95% CI	*pd*	BF
F-F	-0.029	-0.270 – 0.212	0.591	BF01: 3.60 – 7.74 – 16.53
M-F	-0.097	-0.411 – 0.193	0.742	BF01: 1.95 – 5.62 – 9.45
F-M	0.180	-0.117 – 0.456	0.887	BF01: 1.70 – 3.25 – 7.25
M-M	-0.013	-0.431 – 0.395	0.524	BF01: 2.05 – 4.70 – 9.17

Supplementary Table 24 – Within-species contrasts for the z-scored edge weight analysis of dyadic contact aggression count including only interactions between >12 year old individuals.

Contrast		Δ	95% CI	*pd*	BF
			Bonobos		
F-F	M-F	-0.17	-0.52 – 0.21	0.810	BF_{01}: 2.10 – 3.55 – 9.20
F-F	F-M	-0.20	-0.55 – 0.15	0.868	BF_{01}: 1.59 – 3.04 – 6.45
F-F	M-M	-0.34	-0.77 – 0.08	0.941	BF_{10}: 1.28 – 0.71 – 0.40
M-F	F-M	-0.04	-0.43 – 0.34	0.581	BF_{01}: 2.10 – 4.90 – 8.59
M-F	M-M	-0.18	-0.63 – 0.28	0.777	BF_{01}: 1.30 – 3.27 – 5.23
F-M	M-M	-0.14	-0.58 – 0.31	0.720	BF_{01}: 1.50 – 3.55 – 6.39
			Chimpanzees		
F-F	M-F	-0.24	-0.41 – -0.04	0.990	BF_{10}: 4.28 – 1.51 – 0.99
F-F	F-M	0.00	-0.18 – 0.19	0.517	BF_{01}: 4.21 – 10.36 – 19.51
F-F	M-M	-0.32	-0.55 – -0.10	0.999	BF_{10}: 26.55 – 7.84 – 3.79
M-F	F-M	0.24	0.05 – 0.42	0.992	BF_{10}: 3.79 – 1.74 – 0.92
M-F	M-M	-0.09	-0.31 – 0.12	0.798	BF_{01}: 2.65 – 6.65 – 14.74
F-M	M-M	-0.33	-0.54 – -0.11	0.998	BF_{10}: 19.16 – 7.64 – 2.90
